# Supplementary material for: Arabidopsis 3β-Hydroxysteroid Dehydrogenases/C4-Decarboxylases Are Essential for the Pollen and Embryonic Development
Source: Int J Mol Sci. 2023 Oct 25;24(21):15565. doi: 10.3390/ijms242115565 (PMC10649741; doi:10.3390/ijms242115565)
Supplement: Supplementary file 1 [file ijms-24-15565-s001.zip › ijms-2668693-supplementary.pdf]

**Supplementary Table S1**. The oligonucleotides for generating guide RNAs.

| Gene target | Guide RNAs | Oligonucleotides sequence (5'-3') |
|---|---|---|
| *HSD1* | *sgRNA-DT1* | TTGTGTTTGACGGGGTCCA TGG |
| | *sgRNA-DT2* | CTAGCAGCATATTTGGTCC TGG |
| *HSD2* | *sgRNA-DT3* | TCGACGAAGGATTAAGATC TGG |
| | *sgRNA-DT4* | GCGGAGGTGGTGTTTCATA TGG |

**Supplementary Table S2**. Sequence of primers used in this study.

| Primer Name | Sequence (5'-3') | Usage |
|---|---|---|
| *HSD1-DT1-BsFor* | ATATATGGTCTCGATTGTTGTGTTTGACGGGGTCCAGTTTTAGAGCTAGAAATAGC | These 6 primers are for generating the |
| *DT0-BsR2-Rev* | ATATTATTGGTCTCAATCTCTTAGTCGACTCTACCAAT | *CRISPR/Cas9-HSD1/2* transgene |
| *HSD1-DT2-BsFor* | ATATTATTGGTCTCAAGATTGCTAGCAGCATATTTGGTCCGTTTTAGAGCTAGAAATAGC | |
| *DT0-BsR3-Rev* | ATATTATTGGTCTCATCACTACTTCGTCTCTAACCAT | |
| *HSD2-DT3-BsFor* | ATATTATTGGTCTCAGTGATTGTCGACGAAGGATTAAGATCGTTTTAGAGCTAGAAATAGC | |
| *HSD2-DT4-BsRev* | ATTATTGGTCTCTAAACTATGAAACACCACCTCCGCCAATCACTACTTCGACTCTAGCTGTAT | |
| | | |
| *Cas9-For* | ATCCAATCTTCGGCAACAT | Used for screening the transgene-free |
| *Cas9-Rev* | TATCCAGGTCATCGTCGTA | *hsd1/hsd2* mutants |
| *HSD1-For* | AATCACCAGCTTCAGTACTC | For identifying the *hsd1* mutations by |
| *HSD1-Rev* | ACCAGAGCATAATAGGTCAC | AvaII digestion of the PCR fragments |
| *HSD2-For* | CATTACACACCAAAGCAGTG | For identifying the *hsd2* mutations by |
| *HSD2-Rev* | CCAACATCAACACAAGCATC | gel separating the PCR fragments |
| *pLAT52-HSD2-For* | TTACCATCGCGAGCGGTACCATGTCGCCGGCAGCTACGGA | For generating the *pLAT52::HSD2-* |
| *pLAT52-HSD2-Rev* | AGGTCGACTCTAGAGGATCCGTCATGTTTCTTGCTTCCGA | *FLAG* transgene |
